# Hepatitis B Virus HBx Protein Mediates the Degradation of Host Restriction Factors through the Cullin 4 DDB1 E3 Ubiquitin Ligase Complex

**DOI:** 10.3390/cells9040834

**Published:** 2020-03-30

**Authors:** Marissa M. Minor, F. Blaine Hollinger, Adrienne L. McNees, Sung Yun Jung, Antrix Jain, Joseph M. Hyser, Karl-Dimiter Bissig, Betty L. Slagle

**Affiliations:** 1 Department of Molecular Virology and Microbiology, Baylor College of Medicine, Houston, TX 77030, USA; mm3@bcm.edu (M.M.M.); blaineh@bcm.edu (F.B.H.); amcnees@bcm.edu (A.L.M.); jh126641@bcm.edu (J.M.H.); 2 Dan L. Duncan Cancer Center, Baylor College of Medicine, Houston, TX 77030, USA; syjung@bcm.edu; 3 Department of Biochemistry, Baylor College of Medicine, Houston, TX 77030, USA; 4 Mass Spectrometry Proteomics Core, Baylor College of Medicine, Houston, TX 77030, USA; antrixj@bcm.edu; 5 Center for Cell and Gene Therapy, Baylor College of Medicine, Houston, TX 77030, USA; karldimiter.bissig@duke.edu

**Keywords:** hepatitis B virus, HBx, damaged DNA binding protein 1, cullin 4 RING E3 ligase, SMC6, DDB1 cullin accessory factor

## Abstract

The hepatitis B virus (HBV) regulatory HBx protein is required for infection, and its binding to cellular damaged DNA binding protein 1 (DDB1) is critical for this function. DDB1 is an adaptor protein for the cullin 4A Really Interesting New Gene (RING) E3 ubiquitin ligase (CRL4) complex and functions by binding cellular DDB1 cullin associated factor (DCAF) receptor proteins that recruit substrates for ubiquitination and degradation. We compared the proteins found in the CRL4 complex immunoprecipitated from uninfected versus HBV-infected hepatocytes from human liver chimeric mice for insight into mechanisms by which HBV and the cell interact within the CRL4 complex. Consistent with its role as a viral DCAF, HBx was found in the HBV CRL4 complexes. In tissue culture transfection experiments, we showed that HBx expression led to decreased levels of known restriction factor structural maintenance of chromosomes protein 6 (SMC6) and putative restriction factors stromal interaction molecule 1 (STIM1, zinc finger E-box binding homeobox 2 (ZEB2), and proteasome activator subunit 4 (PSME4). Moreover, silencing of these proteins led to increased HBV replication in the HepG2-sodium taurocholate cotransporting polypeptide (NTCP) infection model. We also identified cellular DCAF receptors in CRL4 complexes from humanized mice. Increasing amounts of HBx did not reveal competitive DCAF binding to cullin4 (CUL4)-DDB1 in plasmid-transfected cells. Our results suggest a model in which HBx benefits virus replication by directly or indirectly degrading multiple cellular restriction factors.

## 1. Introduction

Chronic infection with hepatitis B virus (HBV) affects over 257 million people worldwide [1] and is a leading risk factor for the development of serious liver disease, including cirrhosis and hepatocellular carcinoma (HCC). The currently approved antiviral treatments (pegylated interferon-α; nucleos(t)ide analogs) reduce levels of viral replication (reviewed in Reference [2]). A cure for chronic HBV remains elusive, in part, because of an inability to eliminate HBV covalently closed circular (ccc) DNA, a replicative intermediate that serves as the template for viral transcription. Novel strategies are needed to disrupt chronic HBV replication and will require new insight on how HBV interacts with a host cell to sustain viral replication.

The 3.2-kB HBV genome encodes the polymerase (*P*), core (*C*), surface (*S*), and *X* genes. The 17-kD HBx protein product of the *X* gene is of particular interest because it is required for HBV infection in human liver chimeric mice [3] and in HepaRG cells [4] and is required for maximal virus replication in the HBV plasmid DNA model of virus replication [5,6] (reviewed in Reference [7]). However, the functions of HBx in the virus life cycle are not completely understood. As the sole HBV regulatory protein, HBx has multiple functions including transactivation of viral and cellular promoters [8,9], binding to cccDNA and modifying its epigenetic regulation [10,11], and targeting for degradation cellular factors that restrict virus replication [12,13,14]. HBx is reported to interact with over 100 cellular proteins [15], consistent with HBx effects on diverse pathways such as calcium signaling, cell cycle progression, and apoptosis (reviewed in References [7,16,17,18]).

**Figure 1:**
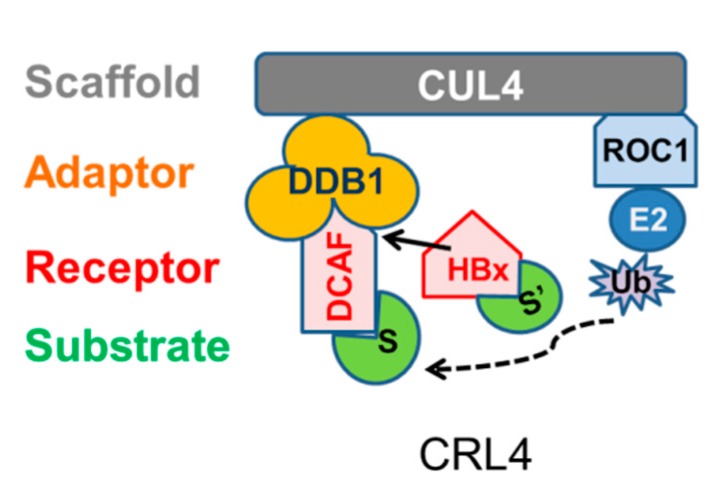
Cullin-4 RING ubiquitin ligase complex (CRL4): The CRL4 complex has a modular structure consisting of the cullin-4A (CUL4) scaffold, the damaged DNA binding protein 1 (DDB1) adaptor protein, and the DCAF receptor (DCAF) proteins that recruit substrate (S) proteins for ubiquitination and degradation. The crooked S’ indicates different substrates recruited by HBx. The regulator of cullins protein 1 (ROC1) contains a Really Interesting New Gene (RING) finger domain, which binds the ubiquitin-charged E2 (ubiquitin conjugating enzyme) [36]. The hepatitis B virus (HBV) HBx protein is a viral DCAF that binds to DDB1 [24,25] via a motif shared with cellular receptors [26,30] (reviewed in Reference [34]). In the present study, the CRL4 complexes from uninfected and HBV-infected human hepatocytes were isolated and the associated proteins were identified and compared to provide insight into the impact of HBV replication on the CRL4 complex. Figure is modified with the publisher’s permission [37].

HBV and many other viruses exploit host cellular ubiquitin machinery for their own benefit (reviewed in References [19,20,21,22,23]). It has been reported that HBx binds to the cullin 4 RING E3 ubiquitin ligase complex (CRL4) via its interaction with the CRL4 adaptor protein damaged DNA binding protein 1 (DDB1) (Figure 1) [24,25,26]. The HBx-DDB1 interaction is conserved among mammalian hepadnaviruses [25] and is required for virus infection and replication in woodchucks [27] and for maximal replication in the HBV plasmid replication assay [28,29]. As a CRL4 adaptor protein, DDB1 mediates its function through interactions with DDB1 cullin-associated factor (DCAF) receptors that recruit specific substrates to the CRL4 for ubiquitination, and this generally, although not always, results in proteasomal degradation [30,31,32] (reviewed in Reference [33]) (Figure 1). Such substrates include the HBV restriction factors Structural Maintenance of Chromosomes Protein 5 and 6 (SMC5/6), discovered by binding to a DDB1-HBx fusion protein [13] and by tandem affinity purification from HepG2-HBx cells [14]. Existing data support the idea that HBx is a viral DCAF, including that HBx contains a DDB1-binding motif shared among other DCAF proteins [26] (reviewed in Reference [34]); it competes with DCAFs DDB2 and DCAF9 for binding to DDB1 [26,35], and it is stabilized by its interaction with DDB1 rather than being ubiquitinated and degraded [29].

Several viruses encode proteins that specifically interact with the CRL4 complex (Table 1) (reviewed in Reference [38]), one of over 200 distinct CRLs in human cells (reviewed in Reference [39]). These virus–cell interactions result in degradation of critical innate immune response proteins [40,41], disruption of the cell cycle [42,43], and degradation of viral restriction factors [12,13,14] (Table 1). Identifying the critical function(s) provided by the HBx–DDB1 interaction has been difficult, in part due to the lack of convenient and authentic HBV infection models in cell culture. Recent advancements with sodium-taurocholate cotransporting polypeptide (NTCP)-overexpressing HBV culture systems have facilitated viral infection studies. However, the host gene expression profile is significantly altered in cancer cell lines compared to primary human hepatocytes in vivo [44]. Human liver chimeric mice have been used for many years to study hepatitis virus replication [45,46,47,48]. In this study, we utilize human hepatocyte tissue from the same donor in an infectious and normal setting.

The goal of our study was to investigate the impact of HBV infection on the CRL4 complex in order to gain insight into the contribution of the HBx–DDB1 interaction to virus replication. HBV infection in human liver was studied in the *FAH^−/−^*, *RAG2^−/−^*, and *IL2RG^−/−^* mouse model in which human liver chimeric mice were infected with HBV subtype *ayw*. The CRL4 was purified from the livers of uninfected (CRL4^UN^) and HBV-infected (CRL4^HBV^) human liver chimeric mice, and the CRL4-associated proteins were identified by mass spectrometry (MS). We tested two hypotheses generated by these data sets. In the first, we characterized four proteins unique to CRL4^UN^ as putative HBV restriction factors using in vitro transfection experiments and restriction factor silencing in the HepG2-NTCP HBV infection model. In the second, we identified six DCAF receptor proteins present in both CRL4^UN^ and CRL4^HBV^ and tested the ability of HBx to compete with different DCAFs for binding to CUL4-DDB1. Overall, our results suggest that multiple cellular restriction factors are present that limit HBV and that one of the functions of HBx may be to direct these proteins for CRL4-mediated degradation, thereby increasing virus replication.

## 2. Materials and Methods

### 2.1. Ethics Statement

Approval for all experiments involving mice was obtained from the Institutional Animal Care and Use Committee at Baylor College of Medicine (IACUC Protocols AN-346 and AN-6272) and the recommendations in the Guided for the Care and Use of Laboratory Animals (Institute for Laboratory Animal Research, National Research Council, National Academy of Sciences, 2011). Human hepatocytes used for transplantation into mice were from cadavers and are not considered human subject research.

### 2.2. Human Liver Chimeric Mice and HBV Infection

Human liver chimeric mice (*FAH^−/−^*, *RAG2^−/−^*, and *IL2RG^−/−^*) were repopulated with cadaveric hepatocytes as previously described [48,58]. Human hepatocytes were enriched in the murine liver by applying selection pressure against the murine hepatocyte upon withdrawing the small molecule inhibitor NTBC (Nitisinone [47]). Mice were put back on the drug (cycling of NTBC) to avoid graft failure as described earlier [47]. After three months, human albumin (ELISA, Bethyl Laboratories) was measured in the murine blood to determine human chimerism. Six mice with similarly high human chimerism (>1 mg/mL human albumin) were selected for this study so that animal-to-animal variation in repopulation was not a variable. Human liver chimeric mice were injected via tail vein with 1 × 10^8^ genome equivalents (GE) of chimpanzee-derived HBV (subtype *ayw*), using the same conditions (mice, inoculum) that have led to nearly 100% of human hepatocytes infected by HBV in our previous work [48]. Blood samples were obtained by retro-orbital bleed 10 min postinfection and weekly until infected mice reached a plateau in viremia. Based on our experience with nearly 100 similarly HBV-infected mice, the viremia reaches a plateau within a few weeks and maintains a relatively constant level of viremia with usually less than 1 log variation.

### 2.3. Real-Time qPCR Quantification of HBV Capsid-Associated DNA

Capsid-associated HBV DNA was extracted from 25 μL of cell media or from 12.5 µL serum using the Kingfisher Flex purification system and the 5× MagMax Pathogen RNA/DNA kit (Biosystems Thermo Fisher Scientific) following the manufacturer’s protocol. Viral DNA was quantified by Taqman qPCR and compared to plasmid standards diluted 10^0^ to 10^7^ as previously described [59].

### 2.4. Immunoprecipitation (IP)/MS

IP/MS analysis of CRL4 complexes was performed by the Mass Spectrometry-Based Proteome Profiling Core at Baylor College of Medicine. Uninfected and HBV-infected human liver chimeric mice (3 each) were sacrificed at maximal viremia. Livers were perfused with Krebs-Ringer solution and livers were then lysed in sodium choride, ethylenediaminetetraacetic acid (EDTA), Tris, nonyl phenoxypolyethoxylethanol (NP-40) (NETN) buffer (50 mM Tris-HCl pH 7.5, 1 mM EDTA, 150 mM KCL, and 0.5% NP-40) containing a protease inhibitor cocktail (P3100, GenDepot) and sonicated for 3 min on ice. Since the goal of this MS screen was to identify new restriction factors, we separately pooled uninfected and HBV-infected liver lysates to ensure that the pulldown was technically reproducible. Two independent IPs were performed. Following centrifugation, the supernatants were IP’d with rabbit anti-CUL4A (Supplementary Table S1). In some experiments, purified protein complexes captured on protein A beads were alkylated before SDS-PAGE to reduce disulfide bonds. Complexes were incubated in 100 µL of 5 mM dithiothreitol (DTT) in 100 mM ammonium bicarbonate and for 45 min at 56 °C. Iodoacetamide was added to 14 mM final concentration and incubated for 30 min at room temperature in the dark to alkylate cysteines [60]. Excess iodoacetamide was quenched by adding 5 mM DTT and by incubating 15 min at room temperature in the dark. Alkylated samples were loaded onto NuPage 10% Bis-Tris midi-gels (Thermo Fisher WG1201BX10) and electrophoresed at 80 V constant voltage. The SDS-polyacrylamide gel was minimally stained with Coomassie brilliant blue. The lane was then cut into 4 pieces and subjected to in-solution digestion with 100 ng of trypsin in 50 mM ammonium bicarbonate (MS grade GenDepot) overnight. Digested peptides were extracted with 350 µL of 100% acetonitrile and 20 µL of 2% formic acid and dried in a Savant Speed-Vac. Dried peptides were dissolved in 10 µL of loading solution (5% methanol containing 0.1% formic acid) and subjected to a nano-HPLC 1000 system (Thermo Scientific) coupled to an LTQ Orbitrap Elite™ (Elite) mass spectrometer. An in-house 2 cm × 100 cm inner diameter trap column with 3 µM Reprosil-Pur Basic C18 beads (Dr. Maisch HPLC GmbH, Germany) and an in-house 5 cm × 150 µM capillary column packed with 1.9 µM Reprosil-Pur Basic C18 beads were used to enrich and separate injected peptides. A 75 min discontinuous gradient of 2–24% acetonitrile, 0.1% formic acid at a flow rate of 800 nL/min was applied to the column and then electro-sprayed into the mass spectrometer. The instrument was operated under the control of Xcalibur software Version 2.2 (Thermo Fisher Scientific) in data-dependent mode, acquiring fragmentation spectra of the top 25 strongest ions. Parent MS spectrum was acquired in the Orbitrap with full MS range of 375–1300 m/z in the resolution of 240,000. Collision-induced dissociation fragmented MS/MS spectrum was acquired in ion-trap with rapid scan mode. Spectral data were then searched against target-decoy human refseq database (release 2015_06, containing 73,637 entries) [61] in Proteome Discoverer 1.4 interface (Thermo Fisher) with Mascot algorithm (Mascot 2.4, Matrix Science). This method identified human-specific peptides but did not distinguish between human versus mouse peptides from highly conserved proteins. Dynamic modifications of acetylation of N-term, alkylation of cysteine, and oxidation of methionine were allowed. The precursor mass tolerance was confined within 20 ppm with fragment mass tolerance of 0.5 Dalton, and a maximum of two missed cleavages was allowed. Assigned peptides were filtered with 1% false discovery rate (FDR) using Percolator validation based on q-value.

### 2.5. Quantitation of MS Data

The intensity-based absolute quantification (iBAQ) number provides a measure of the relative abundance of each protein identified in an IP/MS sample and was calculated as the sum of the intensities of all tryptic peptides for a given protein divided by the number of theoretically observable peptides for that protein [62]. All protein iBAQ numbers were normalized to the CUL4 iBAQ number from the same IP reaction to permit comparison of relative protein levels in the absence versus presence of HBV. The function VLOOKUP (Microsoft Excel) was used to detect overlap between proteins from CRL4^UN^ vs. CRL4^HBV^.

### 2.6. Plasmid DNAs

Two human influenza hemagglutinin (HA) epitope tags (YPYDVPDYA) were added to the N-terminus of HBx by PCR cloning to create pSI-2HA-X [59]. Similarly, two N-terminal HA tags were added to codon-optimized DCAF11 (NCBI NM_001163484.1), DCAF8 (NCBI NM_015726.2), and cereblon (CRBN) (NCBI NM_016302.3), which were then cloned into the NotI and EcoRI sites of the pSI vector. The myc-tagged CUL4A was obtained from Dr. Yong Cang and was described previously [26]. Codon-optimized NTCP (NCBI 003049.3) was PCR-cloned into the BamHI and XhoI sites of the pLVX-IRES-Hyg lentivirus vector to create pLVX-IRES-Hyg-NTCP. 2HA-X^WT^ was cloned into the EcoRI and BamHI sites of the doxycycline (dox)-inducible pLV-TetOne-Puro vector to create pLV-2HA-X. Two N-terminal HA epitope tags were added to the N-terminus of point mutant HBx proteins HBx^69^, HBx^90/91^, or HBx^R96E^ [63,64] and then cloned into the EcoRI and BamHI sites of the pLV-TetOne-Puro vector.

### 2.7. Plasmids Encoding Short Hairpin RNA (shRNA)

Plasmid DNAs encoding shRNAs directed against proteasome activator subunit 4 (PSME4), zinc finger E-box binding Homeobox 2 (ZEB2), stromal interaction molecule 1 (STIM1), and SMC6 are listed in Supplementary Table S2. Confirmation of shRNA silencing was determined by western blot analysis of plasmid-transfected cells.

### 2.8. Cell Culture and Transfections

Human embryonic kidney (HEK) 293T cells were maintained in Dulbecco Modified Eagle Media (DMEM) supplemented to a final concentration of 10% fetal bovine serum (FBS), penicillin (1000 units/mL final), and streptomycin (1000 μg/mL final). Early passage HepG2 cells obtained from the American Type Culture Collection (ATCC) were maintained in Eagle Modified Essential Media (EMEM) supplemented with 10% FBS, penicillin (1000 units/mL final), streptomycin (1000 μg/mL final), and nonessential amino acids. HepG2-NTCP cells were maintained in DMEM/Ham’F12 (DMEM/F12) media supplemented with 10% FBS, penicillin (1000 units/mL final), streptomycin (1000 μg/mL final), and hygromycin B (250 μg/mL final). HEK293T cells were used for some experiments because they are easily transfected. HEK293T cells were plasmid-transfected in 60-mm plates using polyethylenimine (PEI) (Polysciences Inc.) at a final concentration of 4 μg/mL.

### 2.9. Lentivirus Production and Transduction of Cells

Lentiviruses encoding NTCP, shPSME4, and shZEB2 were prepared by co-transfecting HEK293T cells in 100-mm plates with a plasmid encoding Vesicular stomatitis virus G protein (pVSVG) (Addgene #8454), a plasmid encoding the HIV-1 *gag*, *pol*, *tat*, *and rev* genes (psPAX2) (Addgene #12260), and the appropriate lentivirus plasmid. Lentivirus was harvested 48 hrs posttransfection and qualitatively analyzed using Lenti-X GoStix (Takara Bio USA). Lentiviruses encoding shSTIM1 and shluciferase (LUC) were purchased from the Cell-Based Assay Screening (C-BASS) core facility at Baylor College of Medicine. Stable HepG2-NTCP cells were established by transduction of HepG2 cells with lentivirus encoding NTCP with a final concentration of 8 μg/mL polybrene to facilitate transduction. Lentivirus-containing supernatant was removed after 24 h and fresh media containing 500 μg/mL hygromycin B was added to select for transduced cells. Knockdown cells were established by transduction of HepG2-NTCP cells with the appropriate lentiviruses encoding shRNAs for LUC, ZEB2, PSME4, SMC6, or STIM1, followed by selection with 2 μg/mL puromycin.

### 2.10. Antibodies

The antibodies used for IP, immunofluorescence (IF) staining, immunohistochemistry (IHC) staining, and western blotting are listed in Supplementary Table S1.

### 2.11. IP and Western Blot 

Following transfection, cell lysates were prepared in IP lysis buffer (25 mM Tris-HCl pH 7.4, 150 mM NaCl, 1% NP-40, 1 mM EDTA, and 5% glycerol) and were incubated with the IP antibody at a 1:250 dilution for 1 h at 4 °C and then incubated with staphylococcus Protein A (SPA) or Protein A/G agarose beads (Santa Cruz) for 1 h at 4 °C. The lysates were clarified by centrifugation, and the pelleted SPA/beads were washed three times with cold Phosphate Buffered Saline (PBS). The myc-CUL4A complex was detected in plasmid-transfected cells by IP with mouse anti-C-myc using Protein A/G agarose beads (Santa Cruz). Laemmli buffer was added and the SPA/beads were boiled at 95 °C for 5 min, centrifuged, and loaded onto 4–20% gradient (for detection of HBx) or 10% (for all other proteins) mini-Protean Tris-Glycine-Extended (TGX) polyacrylamide gels (Bio-Rad). After separation by SDS-PAGE, proteins were transferred to nitrocellulose membranes. Membranes were blocked with Odyssey Blocking Buffer-Tris Buffered Saline (TBS) (LI-COR) for 1 h at room temperature and incubated with primary antibody with TBS containing 0.1% Tween-20 (TBS-T) overnight at 4 °C. Membranes were washed with TBS-T and incubated with secondary antibody for 1 h at room temperature. TBS was used to wash the membranes three times before imaging on a LI-COR Odyssey CLx.

### 2.12. Western Blot Normalization and Quantitation

Proteins within a lane were normalized to either total protein (REVERT Total Protein Stain, LI-COR) or to a housekeeping protein (LI-COR Western Blot Normalization Handbook). Western blots were quantitated using Image Studio Software (LI-COR). All westerns were reproduced in at least three independent experiments.

### 2.13. HBV Infection of HepG2-NTCP Cells 

Cells were seeded in 96-well plates to be 90% confluent the following day. polyethylene glycol (PEG)-concentrated HBV derived from HepG2.2.15 cells was used as inoculum. Cells were infected with 1000 GE/cell in media containing final concentrations of PEG (4%), DMSO (2.5%), and DMEM/F12 (3% FBS). The cells were spinoculated at room temperature for 1 h at 1000 × *g* to enhance virus infection [65]. The following day, the inoculum was removed and the cells were washed three times with PBS. Replication media containing DMEM/F12 media with 3% FBS and 2.5% DMSO was added back to the cells, and the media changed every 2–3 days until the cells were fixed at seven days postinfection. In some experiments, cells were treated with HBV entry inhibitor Myrcludex B (MyrB; 500 nM) for 30 min prior to and during infection, as described [66,67].

### 2.14. Detection and Quantitation of HBV Core Protein by IF

Uninfected or HBV-infected HepG2-NTCP cells were fixed with 4% formaldehyde for 30 min, permeabilized with TBS containing 0.5% Triton X-100 for 10 min and blocked with 5% FBS in PBS for 1 h at room temperature. Cells were incubated with primary antibody overnight at 4 °C. Cells were then washed with PBS and incubated with secondary antibody for 1 hr at room temp. Nuclei were stained with 4′,6-diamidino-2-phenylindole (DAPI) for 5 min at room temperature. Images were captured with a wide field epifluorescence Nikon TiE inverted microscope using a SPECTRAX LED light source (Lumencor) and a phase contrast or a 20× Plan Apo (NA 0.75) differential interference contrast (DIC) objective. Fluorescence and transmitted light images were recorded using an ORCA-Flash 4.0 sCMOS camera (Hamamatsu), and Nikon Elements Advanced Research v4.5 software was used for multipoint position selection, data acquisition, and image analysis. Two methods were used to quantitate the number of core-positive hepatocytes per well. In the first, a 5.8 mm × 5.8 mm stitched image array was acquired using the Nikon Elements Software and then transferred into ImageJ. The number of core-positive cells for the entire area were counted by two individuals (M.M.M. and B.L.S.). In the second method, ten fields (500 µm × 500 µm each) were selected randomly throughout the plate and core positive cells were counted. Following calculations of the number of core-positive cells per well, similar results were obtained from both methods, and subsequent experiments used the second method. The GE value from each well (determined by real-time PCR analysis of the infected cell media) was divided by the total number of core positive cells from the corresponding well to determine GE per infected cell.

### 2.15. Inhibition of CRLs by MLN4924 (Pevonedistat)

HEK293T cells were plated in 60-mm dishes and, the following day, were treated with 0, 0.1, 1, 5, or 10 μM of MLN4924 (MedChemExpress), an inhibitor of the neural precursor cell expressed developmentally down-regulated protein 8 (NEDD8) activating enzyme required for CRL activity [68]. After 24 h of treatment, the cells were rinsed with cold PBS and lysed with 250 μL IP lysis buffer (see above). The lysates were centrifuged at 4 °C at 14,000 rpm for five minutes to pellet cell debris, and the supernatant was analyzed by electrophoresis on 10% SDS-PAGE and western immunoblot, followed by densitometer quantitation.

## 3. Results

### 3.1. Isolation and Characterization of the CRL4 Complex

The interaction of HBx with DDB1 is critical for HBV infection, but the mechanism(s) of action is not completely understood. To investigate the impact of HBV replication on the CRL4, we isolated the CRL4 complex by IP from CRL4^UN^ and CRL4^HBV^ lysates from hepatocytes grown in human liver chimeric mice [48,58]. CRL4-associated proteins were identified by MS (see the Materials and Methods section). This authentic HBV infection model was used to establish the biologic relevance of the identified proteins linked to the HBV infection. The chimeric mice used in this experiment were approximately 80% repopulated with human hepatocytes that were identified within the chimeric liver by anti-FAH IHC staining (Supplementary Figure S1a). Mice were sacrificed when viremia plateaued (Supplementary Figure S1b). Since the anti-CUL4A antibody used for IP reacted with a region of the protein homologous to the highly related CUL4B protein (reviewed in Reference [69]), the CRL complex is referred to hereafter as CRL4.

**Figure 2:**
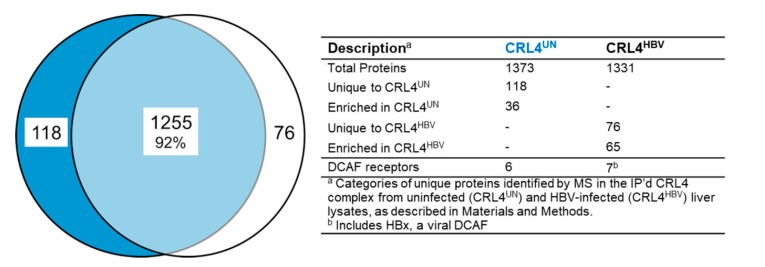
Comparison of CRL4-associated proteins from uninfected and HBV-infected liver tissue: A comparison of the 1373 proteins identified in CRL4^UN^ with the 1331 proteins in CRL4^HBV^ (Supplementary Table S3) revealed that 1255 (92%) of the proteins were present in both complexes. There were 118 proteins found only in the CRL4^UN^ and 76 were only in CRL4^HBV^ (Supplementary Table S4). Proteins identified in each IP were assigned an intensity-based absolute quantification (iBAQ) value (described in the Materials and Methods section) and then normalized to CUL4 levels to determine their relative abundance in the complex. Other proteins were enriched in CRL4^UN^ (n = 36) or the CRL4^HBV^ (n = 65) (Supplementary Table S5a,b). Among the 118 proteins unique to CRL4^UN^ was SMC6, previously described as an HBV restriction factor [13,14].

The number of unique proteins identified in the CRL4^UN^ (n = 1373) was similar to that found in the CRL4^HBV^ (n =1331), and over 92% of the proteins were present in both complexes (Figure 2 and Supplementary Table S3). With nearly 100% of the human hepatocytes infected with HBV [48], we observed that infection did not dramatically alter the overall binding capacity of the CRL4. The presence of the expected CRL4 structural components that includes cullin4A, DDB1, and ROC1 (reviewed in Reference [39]) and of DCAF receptors (described in Figure 1) indicated that the complexes were purified intact. We identified 118 proteins unique to the CRL4^UN^ and 76 proteins unique to the CRL4^HBV^ (Supplementary Table S4). Importantly, the HBx protein was identified in CRL4^HBV^ (Supplementary Figure S2), consistent with its role as a viral DCAF. A cysteine alkylation step was required for the detection of HBx [60]. The relative abundance of each protein in the CRL4 complex was determined by the iBAQ method (see the Materials and Methods section), and all recovered proteins were normalized to the amount of CUL4A in that IP. Proteins were then rank-ordered for their relative abundance within the complex (Supplementary Table S3). Comparison by iBAQ numbers of all proteins in the CRL4 allowed us to identify proteins that were enriched ≥3-fold in CRL4^UN^ versus CRL4^HBV^, and vice versa (Supplementary Table S5).

We initially predicted that proteins unique to CRL4^HBV^ (Supplementary Table S4) or enriched in CRL4^HBV^ (Supplementary Table S5) would include proteins recruited by the virus to the CRL4 complex for ubiquitination and degradation. An additional interpretation was considered after we found the previously described HBV restriction factor SMC6 [13,14] among a subset of 118 proteins that are unique to the CRL4^UN^. Proteins normally processed by the CRL4^UN^ might undergo a more rapid degradation upon HBV infection and might be absent (or reduced) in the CRL4^HBV^. We hypothesized that the group of 118 proteins in the CRL4^UN^ would include additional HBV restriction factors.

### 3.2. HBV Restriction Factors

A screening strategy was developed to determine whether cellular HBV restriction factors, in addition to SMC6, were present in the group of 118 proteins recovered in the CRL4^UN^. Steps in this strategy included first establishing that the protein is normally processed through a CRL and then demonstrating that HBx expression lowers the steady-state levels of the protein. Finally, we determined whether silencing of the putative restriction factor led to elevated HBV replication. Five proteins that were unique to CRL4^UN^ were selected for initial studies as potential restriction factors based on their potential to restrict the HBV life cycle. For example, Proteasome Activator Subunit 4 (PSME4) is reported to mediate the degradation of acetylated histones [70,71], and acetylated histones play a role in the transcriptionally active form of viral cccDNA [72]. Zinc Finger E-Box Binding Homeobox 2 (ZEB2) overexpression inhibits HBV replication in HepG2.2.15 cells through an interaction with the core promoter [73], but its interactions with HBx or the CRL4 are unknown. Stromal Interaction Molecule 1 (STIM1) is an endoplasmic reticulum calcium sensor that regulates extracellular calcium entry into the cytoplasm [74]. Elevated calcium benefits HBV replication [75,76], but the mechanisms exploited by HBV to modulate this increased cytosolic calcium to prevent calcium overload and subsequent apoptosis are unknown. Proteasome Activator Subunit 1 (PSME1) is a subunit of the immunoproteasome (reviewed in Reference [77]) that generates antigenic peptides for presentation on MHC class I molecules and may be important in the host response to HBV. SMC6 was included in the screening analysis because its interaction with CRL4 in the presence of HBx has been previously studied [13,14].

**Figure 3:**
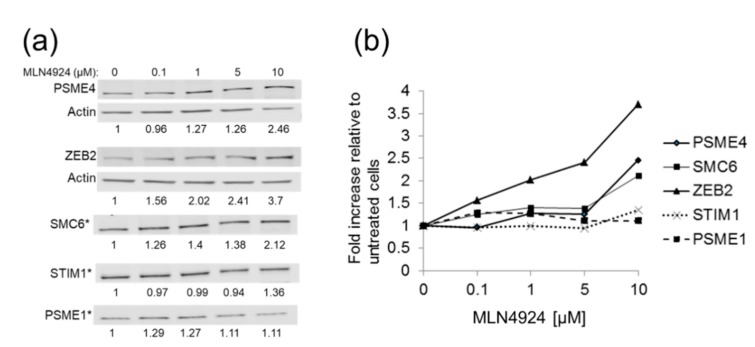
Putative HBV restriction factors are processed through a CRL complex: (**a**) HEK293T cells were cultured for 24 h in the presence of increasing concentrations of the CRL inhibitor MLN4924. Protein levels for putative restriction factors PSME4, Zinc Finger E-Box Binding Homeobox 2 (ZEB2), STIM1, and PSME1 and for SMC6 [13,14] in the absence and presence of MLN4924 were determined by western blot assays and normalized either to total protein (indicated by asterisks) or to actin (described in the Materials and Methods section). (**b**) Graphical representation of western blot shown in panel (**a**): The level of normalized restriction factor in untreated cells was set to 1.0 and the levels of the same protein in MLN4924-treated cells compared to that. PSME4, ZEB2, and SMC6 showed increasing levels with increasing amounts of MLN4924, indicating they are normally processed through a CRL. STIM1 levels increased with the highest dose (10 μg), while PSME1 levels were not impacted by drug treatment. Results shown are representative of n = 3 independent experiments.

To confirm that the putative restriction factors were processed through a CRL, HEK293T cells were cultured in the presence of increasing concentrations of MLN4924 (Pevonedistat), a small molecule inhibitor of the NEDD8-activating enzyme that is required for CRL function [68]. If the candidate restriction factor was normally processed through a CRL, its steady-state level should increase in the presence of increasing concentrations of MLN4924. Indeed, increased steady-state levels of PSME4, SMC6, and ZEB2 were reproducibly detected with increasing amounts of MLN4924 (Figure 3). STIM1 protein levels increased only when cells were treated with the highest dose (10 μM) of MLN4924 (Figure 3b). Since MLN4924 had little effect on PSME1 levels (Figure 3), this protein was not included in further experiments. These results confirm that PSME4, SMC6, ZEB2, and STIM1 are normally processed through a CRL.

**Figure 4:**
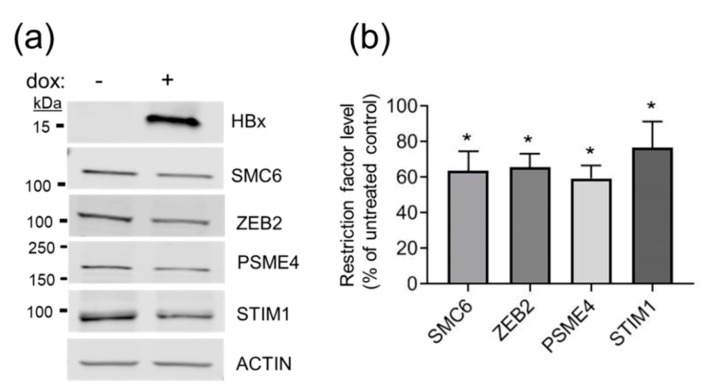
Expression of HBx reduces steady-state levels of putative restriction factors: (**a**) Representative western blot of plasmid-transfected HEK293T cells ± treatment with dox to induce the expression of HBx, as described in the Materials and Methods section. Cell lysates were separated by SDS-PAGE; were transferred to nitrocellulose filters; and were blotted using antibodies against restriction factors SMC6, ZEB2, PSME4, and STIM1 (Supplementary Table S1). Protein levels were normalized to actin levels (bottom panel). Comparison of treated cells with untreated cells revealed the impact of HBx expression. (**b**) Graphical representation of western blot results from three independent experiments: The level of each restriction factor in the absence of HBx was set to 100% and compared to the level of that same protein in the presence HBx. Significance was determined by student *t* test using GraphPad Prism Version 8.1.2. Asterisks indicate *p* < 0.05.

We next examined the impact of HBx expression on the steady-state levels of the putative restriction factors in HEK293T cells, which are easily transfected and are a standard model for protein interaction studies, including a recent study on HBx and SMC5/6 [14]. Cells were transfected with plasmid DNA encoding a dox-inducible HBx and cultured in the presence of dox to induce HBx expression or left uninduced (no dox) to serve as the negative control. HBx expression was associated with a measurable decrease in the steady-state levels of putative restriction factors PSME4, ZEB2, and STIM1 and of SMC6 (Figure 4). The 40% reduction was similar to that previously reported for SMC6 [13,14] and ZEB2 [73] and extends those results to include new candidate restriction factors that may be targeted directly or indirectly by HBx for degradation.

**Figure 5:**
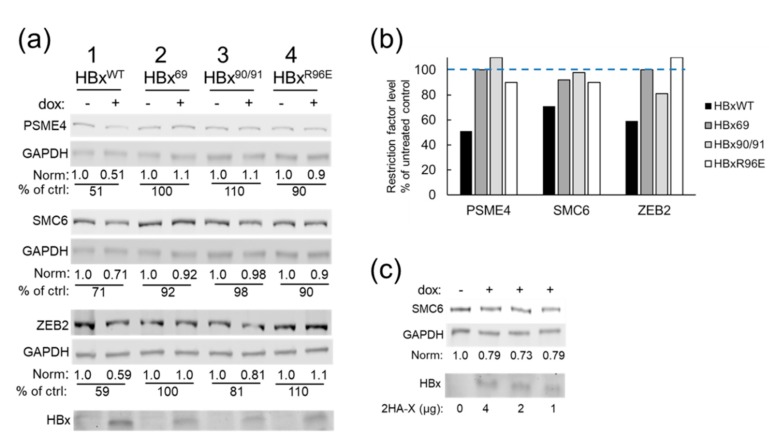
HBx^WT^-DDB1 binding mediates reduced levels of putative restriction factors. (**a**) Representative western blots (n = 3) of lysates from HEK293T cells transfected with plasmids encoding dox-inducible HBx^WT^ or HBx point mutants that do not bind DDB1 (HBx^69^, HBx^90/91^, and HBx^R96E^) [63,64]. Following SDS-PAGE and western blot analysis, protein levels were normalized to glyceraldehyde 3-phosphate dehydrogenase (GAPDH). The amount of normalized protein in uninduced cells was set to 1.0 and compared to the normalized level of that protein in the presence of HBx. Steady-state levels of HBx proteins were determined by western blot (bottom panel). (**b**) Graphical representation of western blot results shown in panel (**a**): The level of each restriction factor in the absence of HBx^WT^, HBx^69^, HBx^90/91^, or HBx^R96E^ was set to 100% and compared to the level of that same protein in the presence of the HBx proteins. (**c**) HEK293T cells were transfected with a control plasmid or decreasing amounts of a plasmid encoding dox-inducible HBx^WT^. After dox-induction, the levels of SMC6 were determined and normalized to GAPDH. The normalized SMC6 level in control cells (i.e., no HBx) was set to 1.0 and compared to dox-treated cells (with HBx). The lowest levels of HBx were comparable to the levels of mutant HBx seen in panel (**a**) lanes 2, 3, and 4 yet were still able to reduce SMC6 levels. Western blot analysis was used to detect the steady state levels of HBx^WT^ (bottom panel).

A previous study reported that HBx expression led to the degradation of SMC5/6 [13,14]. To determine whether an HBx–DDB1 interaction was required for the degradation of the putative restriction factors described above, we next investigated a panel of HBx mutant proteins that do not bind DDB1. SMC6 was included for comparison. Cells were transfected with plasmid DNA encoding dox-inducible wildtype HBx (HBx^WT^) that binds DDB1 or with plasmids encoding point mutant HBx proteins HBx^69^, HBx^90/91^, or HBx^R96E^ that neither bind DDB1 [63,64] nor rescue HBx-deficient replication in a plasmid replication assay [28,29]. While HBx^WT^ was successful at reducing the levels of the putative restriction factors, the point mutant HBx proteins did not (Figure 5a,b). The steady-state levels of the mutant HBx proteins were lower than that of HBx^WT^ (Figure 5a, lower panel), consistent with previous reports that an interaction with DDB1 stabilizes HBx [29,35,78]. To rule out that the lower levels of mutant HBx protein expression was responsible for the failure to decrease the restriction factor levels, cells were transfected with decreasing amounts of plasmid encoding HBx^WT^ (Figure 5c). HBx^WT^ was still able to reduce steady state levels of SMC6 even when present at levels similar to that of HBx mutant proteins (Figure 5c). This result suggests that mutant HBx proteins were present at sufficient levels to reduce the restriction factors if the HBx mutants were functionally capable of this. We note that, while SMC6 completely disappeared from the CRL4^HBV^ (Supplementary Table S3), there was incomplete SMC6 degradation in the whole cell lysates of cells expressing inducible HBx (Figure 5a,c). The different results may be explained by the characteristic protein amounts present in these two methods.

**Figure 6:**
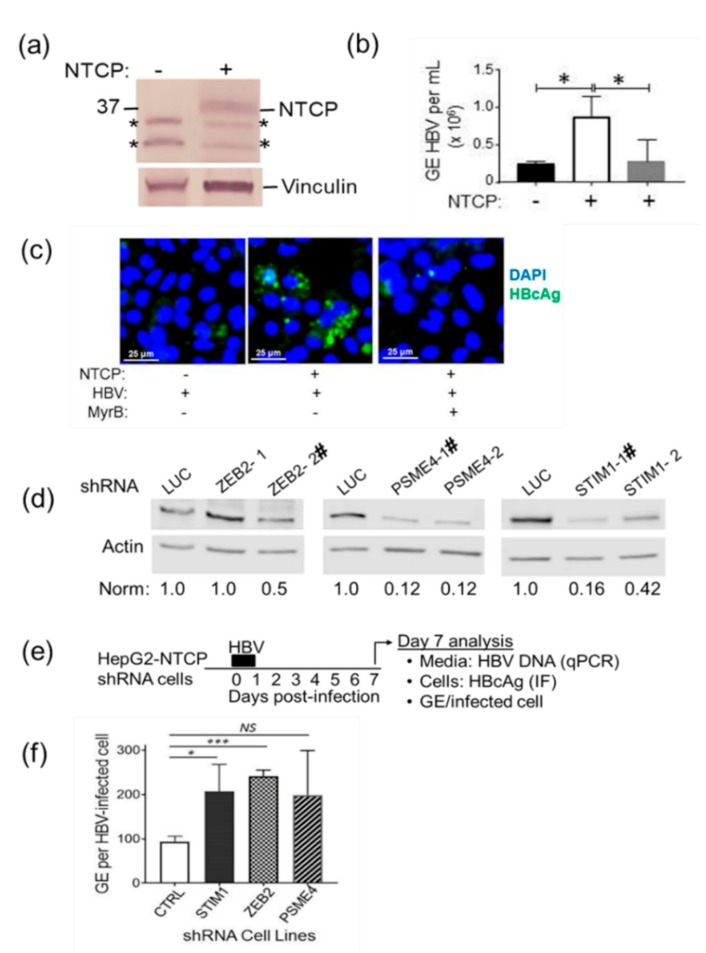
Knockdown of putative restriction factors enhances HBV replication: HepG2 cells overexpressing the HBV receptor sodium-taurocholate cotransporting polypeptide (NTCP) were created as described in the Materials and Methods section. (**a**) Western blot confirmation of NTCP expression: Other bands (indicated by asterisks) are nonspecific. (**b**) HBV DNA was detected in the media of HBV-infected cells as described in the Materials and Methods section and is displayed as genome equivalents (GE) per mL of media. The asterisk (*) indicates *p* < 0.05. (**c**) Immunofluorescence (IF) staining for HBV core antigen (HBcAg) in cells inoculated with HBV: Only cells overexpressing the NTCP receptor are permissive to HBV infection. Pretreatment of cells with HBV entry inhibitor Myrcludex B (MyrB) reduces both the number of cytoplasmic HBcAg-positive cells and the GE/mL (see panel b), indicating that infection occurs through an NTCP pathway [66,67]. (**d**) Western blot of HepG2-NTCP cells transduced with lentivirus encoding shRNAs to knock down levels of ZEB2, PSME4, STIM1, or LUC negative control. Following SDS-PAGE and western blot analysis, levels of normalized restriction factors in control cells were set to 1.0 and compared to normalized levels in cells with restriction factor knockdown. Cell lines designated “**#**” were used for infection experiments. (**e**) Experimental design for data shown in panel (**f**). HepG2-NTCP shRNA cells were infected in triplicate wells with HBV (see the Materials and Methods section). At seven days postinoculation, media was collected to quantitate GE and the cells were fixed and stained with antibody detecting HBcAg to identify infected cells (see the Materials and Methods section). (**f**) Representative results of infection experiments: GE per infected cell were set to 100% for shLUC control cells and compared to levels in the media of shRNA knockdown cells. The increased replication in shZEB cells was reproduced in 3 independent experiments 3 wells per experiment. Increased replication in shSTIM1 or in shPSME4 cells was reproducible in about 75% of experiments but was not always statistically significant due to well-to-well variability. Significance was determined by student *t* test using GraphPad Prism Version 8.1.2. (* *p* < 0.05, *** *p* < 0.001).

Finally, we used the HepG2-NTCP HBV infection model to determine if shRNA targeting of the putative restriction factors would lead to increased HBV replication. HepG2 cells were transduced with lentivirus encoding the HBV receptor NTCP [79] (Figure 6a) to allow permissivity to HBV infection that was lacking in HepG2 cells without NTCP overexpression (Figure 6b). HepG2 cells overexpressing NTCP had significantly elevated levels of HBV replication in comparison to infection of normal HepG2 cells that lacked overexpressed NTCP. Treatment with the HBV inhibitor MyrB significantly reduced the level of HBV replication (Figure 6b,c). Lentiviruses encoding shZEB2, shPSME4, shSTIM1, or shSMC6 were transduced into HepG2-NTCP cells to create stable knockdown cell lines that were used for HBV infection experiments. A lentivirus encoding shLUC was used to create a negative control cell line. Transduction of cells with lentivirus encoding shRNA targeting SMC6 led to cell growth arrest, and so, this cell line could not be included in subsequent analyses. The reason for the growth arrest is unknown but suggests the SMC6 knockdown is not tolerated by these HepG2-NTCP cells. Consistent with this idea, SMC6 knockout is embryonically lethal in mice [80].

We first validated that the shRNA cell lines exhibited lower steady-state levels of their targeted restriction factor (Figure 6d). Next, the cell lines were infected with HBV derived from HepG2.2.15 cells (Figure 6e), and HBV replication was assessed on day 7 by quantifying HBV capsid-associated DNA in the media of infected cells. Relative to HBV replication in the HepG2-NTCP^shLuc^ control cell line, virus replication was reproducibly elevated in all shRNA restriction factor cell lines (Figure 6f). These results are consistent with previous findings that knockdown of ZEB2 in HepG2.2.15 cells leads to increased virus replication [73] and extends those findings into the HepG2-NTCP HBV infection model. The increased virus replication measured in the HepG2-NTCP^shPSME4^ and HepG2-NTCP^shSTIM1^ cells (Figure 6f) reached statistical significance in most experiments. Our results indicate that the knockdown of an individual restriction factor is associated with a reproducible two- to three-fold increase in virus replication. An affect may be due to the proportional silencing of the restriction factor in this cell culture model. It also may indicate that there are additional restriction factors that must be overcome to robustly increase HBV replication.

### 3.3. HBx Does Not Compete with Cellular DCAFs for Binding to DDB1 in the CRL4

A subset of CRL4-associated proteins of great interest to studies of HBV infection is the DCAF receptors that bind DDB1 and recruit substrate proteins to the CRL4 for ubiquitination and degradation (Figure 1). The HBV regulatory HBx protein is a viral DCAF [26,29,35] (reviewed in Reference [34]), but its impact on cellular DCAFs that reside in the CRL4 of human liver is unknown. However, the ability of HBx to bind to the DCAF receptor binding site on DDB1 [26] (reviewed in Reference [34]) raises the possibility that the HBx expressed from the HBV genome during replication may interfere with CRL4-DDB1-DCAF interactions and potentially alter the spectrum of substrates recruited to the CRL4. The human genome encodes approximately 60 DCAF receptors (reviewed in Reference [33]), and 32 of these are detectable by IHC staining in human liver [81,82]. In our study, six of these DCAFs were found in the CRL4^UN^ and in CRL4^HBV^ (Table 2). Three of the six recovered DCAF receptors (CRBN, DCAF11, and DCAF8) were selected for follow-up analysis based on their potential involvement in the HBV life cycle. Their interaction with DDB1 was validated in HepG2 cells by IP of the individual DCAFs, followed by MS detection of DDB1 and CUL4A (Supplementary Table S6). 

We next determined whether these three DCAFs could compete with HBx for binding to DDB1 within the CRL4, since two previous studies suggested that HBx might compete with DCAF receptors for binding to DDB1. In the first, HBx expressed as a GFP-HBx fusion protein competed with DCAF DDB2 in transfected HeLa cells [35]. In the second, peptides spanning the DDB1 binding domain of woodchuck WHx protein competed with DCAF9 for binding to DDB1 in an in vitro pulldown assay [26]. Neither DDB2 nor DCAF9 were found in the CRL4 complexes in our experiments, but we hypothesized that other DCAFs (CRBN, DCAF11, and DCAF8) in the CRL4 from human liver (Table 2) would compete with HBx for binding to DDB1 within CRL4. Current known functions of these DCAFs possibly relevant to the liver are provided (Table 2). Plasmids encoding 2HA-tagged DCAFs were created and tested in transfection experiments to demonstrate that the HA tags did not prevent the DCAFs from entering the CRL4 complex (Supplementary Figure S3). Next, plasmids encoding myc-tagged CUL4A and 2HA-tagged DCAFs were co-transfected into cells in the absence or presence of increasing amounts of plasmid encoding HBx (pSI-2HA-X or control pSI). Transfected cells were detergent-lysed, and the lysates IP’d with an anti-myc antibody to pull down the CRL4 complex, followed by western blot analysis for the presence of DDB1 and the DCAF receptor of interest (Supplementary Figure S4a). In these experimental conditions, increasing amounts of HBx did not alter the ratio of DDB1-CRBN within the CRL4 complex and remained the same as in cells lacking HBx expression. Similar results were obtained with DDB1-DCAF8 and DDB1-DCAF11 (not shown). In summary, we have no evidence that HBx can compete with these DCAFs for binding to DDB1 within the CRL4 and under these experimental conditions.

## 4. Discussion

Viruses have evolved different mechanisms to usurp host cell pathways to benefit virus replication, and the targeting of cellular E3 ligases is a common mechanism used to modify host gene expression (reviewed in References [19,20,38,100]). The HBV HBx protein binding to CRL4 adaptor protein DDB1 is critical for HBV replication [28,29]. However, the mechanism(s) by which this interaction benefits HBV replication is not completely understood. The goal of the present study was to investigate the impact of HBV replication on the CRL4 complex in the authentic and well-established human liver chimeric mouse HBV infection model [48]. Most importantly, we combine in this study the sophisticated animal model with an unbiased MS approach to identify a list of cellular proteins differentially bound to the CRL4 complex. Our studies suggest the existence of multiple cellular restriction factors targeted for inactivation by HBV and indicate that one mechanism by which HBx dampens the expression of these restriction factors is through an interaction between HBx and DDB1.

Interestingly, this study also provides a large database of CRL4-related proteins from normal human liver that can be used by other investigators for hypothesis-driven research. Our initial comparison of CRL4-associated proteins from uninfected versus HBV-infected human liver revealed 118 proteins unique to CRL4^UN^ that were absent in CRL4^HBV^. Since the previously studied HBV restriction factor SMC6 [13,14] was among the 118 proteins, we reasoned that additional restriction factors might also be present in that population of proteins. Indeed, of four additional candidate restriction factors tested in our study, three (PSME4, STIM1, and ZEB2) met the criteria of being processed by a CRL (and stabilized by MLN4924 treatment). They were also present at lower steady-state levels in the presence of HBx. Finally, their silencing led to a reproducible increase in HBV replication in the HepG2-NTCP HBV infection model. Together, these results confirm previous observations that HBx targets SMC5/6 for degradation [13,14] and extends those results to include additional cellular restriction factors that may act to inhibit HBV replication. While previous studies have concluded that a major function of HBx is to direct SMC5/6 for degradation, we now propose an expanded role for HBx in virus replication that involves targeting additional cellular restriction factors.

**Figure 7:**
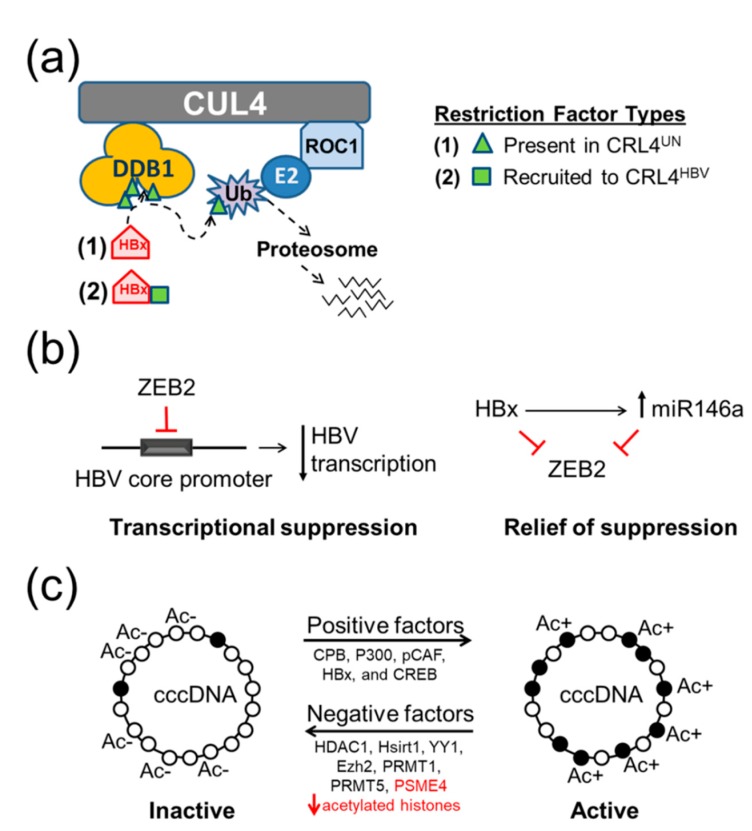
Proposed HBV restriction factor models: (**a**) Based on results of this study, this model proposes at least two categories of HBV restriction factors. Some HBV restriction factors are already present in the CRL4^UN^ (green triangles), while others are recruited to the CRL4 by HBx (green squares). (**b**) Proposed model to explain a role for ZEB2 as an HBV restriction factor. *Left*, ZEB2 is a DNA binding transcriptional repressor previously shown to bind to the HBV core promoter and significantly decrease HBV transcription [73]. *Right*, HBx relieves ZEB2-mediated transcriptional suppression by upregulating miR146a [107], which promotes HBV replication by targeting ZEB2 [106]. In the current study, we propose that HBx utilizes the CRL4 complex to target ZEB2 for degradation. (**c**) Proposed mechanism for restriction factor PSME4. *Left*, Transcriptionally inactive cccDNA has a preponderance of hypoacetylated histones (empty circles) and associates with specific factors ((histone deacetylases (HDACs), transcriptional repressors, and methyltransferases)] such as HDAC1 and Nicotinamide adenine dinucleotide (NAD)-dependent protein deacetylase sirtuin 1 (Hsirt1) [10], yin yang 1 (YY1) and enhancer of zeste homolog 2 (Ezh2) [111], protein arginine methyltransferase 1 (PRMT1) [109], and PRMT5 [110]. *Right*, Alternatively, transcriptionally active cccDNA contains a preponderance of hyperacetylated histones (filled circles) and associates with other factors including cyclic adenosine monophosphate (cAMP) responsive element binding protein 1 (CREB) [108], CREB binding protein (CBP), P300, P300/CBP-associated factor (pCAF), PSME4 (red font, identified in this study) and HBx [10,11]. We propose that PSME4 is a viral restriction factor because of its ability to degrade acetylated histones [70,71], thereby helping to return cccDNA to an inactive state unless HBx is present to promote the degradation of PSME4.

Previous studies demonstrated that HBx-mediated degradation of SMC5/6 requires CUL4 [14] and a form of DDB1 that interacts with CUL4 [13]. It was proposed that HBx serves as a “bridge” to link SMC5/6 to the CRL4 where it is ubiquitinated and degraded [14]. Importantly, we detected SMC6 and other restriction factors in the CRL4 from uninfected liver, indicating that HBx was not needed for their recruitment to the CRL4. Their presence in the CRL4 was supported by demonstrating increased levels of these factors following the addition of MLN4924, a chemical that renders CRLs inactive. Our data supports a model in which there are at least two groups of HBV restriction factors. First, the factors are present and processed by CRL4^UN^ (Figure 7a, green triangles). Upon HBV infection, these factors would be degraded via polyubiquitination as described for SMC5/6 [14]. Alternatively, HBx might mediate a more global effect on restriction factor turnover, perhaps by modulating CRL4 regulatory proteins such as cullin-associated NEDD8-dissociated protein 1 (CAND1) or NEDD8 (reviewed in Reference [101]). In support of this idea, the inhibition of NEDD8 (and of CRL4 activity) by Pevonedistat (MLN4924) also inhibited HBV transcription and restored SMC5/6 levels [102]. The model predicts that a second group of restriction factors are either absent or at low level in CRL4^UN^ but, upon HBV infection, are recruited to the CRL4^HBV^ and present at increased levels (Figure 7a, green squares). Examples of this second group are provided in Supplementary Table S5b and discussed below. Further work is needed to firmly establish these proposed models.

The specific step in virus replication targeted by a cellular restriction factor is expected to vary, depending on the normal function of that cellular protein. For example, SMC6 is part of the highly conserved SMC5/6 complex that binds DNA (reviewed in Reference [103]) and, in HBV-infected cells, is proposed to restrict virus replication by binding to cccDNA to prevent viral transcription [13,14]. In primary human hepatocytes, SMC5/6 localizes to the Nuclear Domain 10 ((also known as promyelocytic leukaemia (PML) body)) where it prevents HBV transcription without initiating an innate immune response [104]. The degradation of SMC5/6 by HBx that ultimately occurs is an evolutionarily conserved function [105]. The results of our study suggest two additional HBV restriction factor models. The first involves ZEB2, a negative regulator of transcription that inhibits HBV through specific binding to the HBV core promoter in the HepG2.2.15 cell model of HBV replication [73] (Figure 7b, left). Relief of this transcriptional repression is mediated through microRNA 146a (miR-146a) [106], and in turn, miR-146a is positively regulated by HBx (Figure 7b, right) [107]. Our finding that HBx mediates the degradation of ZEB2 extends those findings (1) by demonstrating that downregulation of ZEB2 enhances virus replication in the HepG2-NTCP HBV infection model and (2) by suggesting a second mechanism for relief from ZEB2 transcriptional repression, namely through HBx-DDB1-mediated degradation of ZEB2.

A second new HBV restriction factor model arising from our results predicts targeting of HBV cccDNA, which exists in either a transcriptionally active or inactive state, depending upon the associated proteins and the presence of acetylated histones [10,72,108,109,110] (Figure 7c). Cellular PSME4 is a proteasome component that recognizes acetylated histones and targets them for ATP- and ubiquitin-independent degradation during spermatogenesis and the DNA damage response [70,71]. Our model proposes that PSME4 normally cooperates with other cellular factors to return the transcriptionally active cccDNA to the silenced form (Figure 7c). The degradation of PSME4 would allow cccDNA to remain transcriptionally active, thus favoring virus transcription. More research is needed to confirm that STIM1, ZEB2, and PSME4 are indeed restriction factors. For example, they will need to be tested for their ability to rescue HBx-deficient HBV replication in an infection model, as described for the SMC6 restriction factor [13,14]. If confirmed as restriction factors, our results then support the idea that the host cell may restrict HBV replication in multiple processes in virus replication only to be nullified by the HBx–CRL4 interaction. Our initial studies focused on only five of the 118 proteins unique to the CRL4^UN^, and it is likely that additional restriction factors are present in this group of host cell proteins.

A similar CUL4 IP/MS study performed on a transfected human BT474 breast cancer line recovered two DCAF receptors: DCAF11 and human immunodeficiency virus type 1 Viral Protein R (Vpr)-binding protein (VPRBP) [30], of the approximately 60 DCAFs encoded in the human genome (reviewed in Reference [33]). Our finding of six DCAF receptors is a similarly small number. Our results showed that HBx does not compete with CRBN for binding to DDB1 in the CRL4 complex, which contrasts with two previous studies that concluded that HBx competed with the binding of DCAFs DDB2 [35] and DCAF9 [26] to DDB1. However, important differences in experimental design may account for the differing observations. Competition of DDB2-DDB1 with GFP-HBx was demonstrated in HeLa cells transfected with HA-DDB1 and myc-DDB2, followed by IP [35]. In the second study, increasing amounts of woodchuck hepatitis X (WHx) peptide were shown to prevent GST-DCAF9 from copurifying with DDB1 [26]. Neither DDB2 nor DCAF9 were identified in the CRL4 complexes in our study. Importantly, the previous studies did not examine the impact of HBx on CUL4-bound DDB1-DCAF, as was done in the current study.

A subset of proteins not pursued in our study includes those unique to CRL4^HBV^ (n = 76) and/or enriched in CRL4^HBV^ (n = 65). Proteins in these groups could be cellular proteins targeted by HBx or other HBV proteins for recruitment to and degradation by the CRL4. Alternatively, they may be upregulated as a normal response to viral infection and thus appear in the CRL4^HBV^. Data from other laboratories support both of these scenarios. For example, included as a protein unique to CRL4^HBV^ is DEAD box protein 5 (DDX5), an RNA helicase that has an emerging regulatory role in viral infections, including HBV (reviewed in Reference [112]). DDX5 was identified as an HBx interacting protein in HepG2-HBx cells that were treated with MLN4924 [14]. Previous studies showed that HBV infection or expression of HBx alone caused downregulation of DDX5, preventing its ability to regulate the stability and function of the chromatin modifying polycomb repressive complex and leading to increased viral transcription [113]. The finding of DDX5 in the population of proteins unique to CRL4^HBV^ is consistent with it being recruited to the CRL4 for degradation and supports the idea that DDX5 downregulation can occur through the CRL4 pathway. A second cellular protein of interest is Protein Phosphatase 2 Catalytic Subunit Alpha (PPP2CA), also previously identified as an HBx-interacting protein [114]. HBx inhibits PPP2CA phosphatase activity and associated cell death. With PPP2CA recruited to the CRL4^HBV^ complex for degradation, the survival of infected cells that might normally undergo apoptosis could contribute to HBV-associated hepatocarcinogenesis [114]. A final example is the RUVBL2 component of the nucleosomal acetyltransferase of the histone H4 (NuA4) complex [115]. This complex binds to the HBV precore/core promoter to inhibit HBV transcription, and knockdown of RUVBL2 leads to increased HBV replication [115]. Our finding of RUVBL2 enriched in CRL4^HBV^ (Supplementary Table S5b) suggests that a mechanism of RUVBL2 downregulation involves a CRL4 pathway.

## 5. Conclusions

This study investigated the impact of HBV infection and replication on the CRL4 complex isolated from human hepatocytes in vivo. Using an authentic virus infection model and a sophisticated CUL4 IP/MS approach, we identified a list of potential host HBV restriction factors that led to testable hypotheses. Our results suggest that HBx interacts with the CRL4 complex in a DDB1-dependent manner to play an intricate role in altering host cell protein levels, which leads to enhanced virus replication. Because a high viral load is a significant predictor of the development of HCC, this observation may have clinical relevance. The unbiased comparison of CRL4-associated proteins from uninfected and HBV infected human hepatocytes provides a rich dataset from which testable hypotheses can be generated.

## Figures and Tables

**Table 1:** Viral proteins that interact with CUL4-DDB1: Proteins encoded by several viruses interact directly with CUL4-DDB1 and benefit virus replication. For example, several paramyxoviridae express V proteins that target signal transducer and activator of transcription (STAT) proteins for degradation via CUL4-DDB1 to inactivate the innate immune response. Other viral proteins and the cellular functions targeted are also listed. Table modified with permission from Reference [19].

Virus Family	Virus (Protein)	Pathway Affected	Reference
Paramyxoviridae	SV5 ^a^ (V)	Innate immunity	[41]
	HPIV2 ^b^ (V)	Innate immunity; cell cycle	[49]
	Mumps (V)	Innate immunity	[40]
Hepadnaviridae	HBV ^c^ (HBx) WHV ^d^ (WHx)	Degrades SMC5/6 Unknown	[13,14]
Retroviridae	HIV-1 ^e^ (Vpr)	Cell cycle	[42,50,51]
	HIV-2 ^f^ (Vpx)	Cell cycle	[52]
Flaviviridae	HCV ^g^ (BS3/4A)	Cleaves DDB1	[53]
Herpesviridae	M-γHV68 ^h^ (M2)	Inhibits apoptosis	[54]
	EBV ^i^ (BPLF1)	Cell cycle	[55]
	Bovine Herpes (VP8) MCMV ^j^ (pM27)	Unknown Innate immunity	[56] [57]

^a^ SV5, simian virus 5; ^b^ HPIV2, human parainfluenza virus 2; ^c^ HBV, hepatitis B virus; ^d^ WHV, woodchuck hepatitis virus; ^e^ HIV-1, human immunodeficiency virus type 1; ^f^ HIV-2, human immunodeficiency virus type 2; ^g^ HCV, hepatitis C virus; ^h^ M-γHV68, murine gamma herpesvirus 68; ^i^ EBV, Epstein-Barr virus; ^j^ MCMV, mouse cytomegalovirus.

**Table 2:** DCAF receptor proteins identified in the CRL4 of human liver: Six DCAF receptor proteins were identified in the CRL4 complex from both uninfected and HBV-infected human hepatocytes. HBx, a viral DCAF receptor [26,29,83] (reviewed in Reference [34]), was also identified in the CRL4 from HBV-infected human hepatocytes.

DCAF ^a^	Alias ^b^	Functions Possibly Relevant to the Liver ^c^
DCAF11	WDR23	Mediates degradation of stem-loop binding protein [84], histone mRNA maturation [84,85]
DCAF8	WDR42A	Targets histone H3K79 for polyubiquitination [86], p.R317C mutation results in decreased DDB1–DCAF8 association [87]
Cereblon	CRBN	Degradation of MEIS2 [88], functions as Lon-type protease in mitochondria [89]
PAFAH1B1	LIS1	Reduced mRNA and protein levels in 70% of hepatocellular carcinoma tissues [90], hepatic deletion induces fatty liver and increases genomic instability and tumorigenesis [91], involved in dynein motor complex [92,93,94,95]
RBBP4 ^d^	RBAP48	Subunit of the nucleosome remodeling and histone deacetylase (NuRD) complex [96]
WDR26 ^d^	CDW2	Negative regulator of Wnt signaling pathway [97]
HBx ^d^	-	Required for virus replication [3,4], co-factor in development of hepatocellular carcinoma [98,99]

^a^ DCAF, DDB1 CUL4 Accessory Factor; ^b^ Alias, alternative name for the same protein; ^c^ Described DCAF functions that may be relevant to the liver; ^d^ Alkylation step prior to mass spectrometry analysis was needed in order to detect RBBP4, WDR26, and HBx.

## Supplementary Materials

The following are available online at https://www.mdpi.com/2073-4409/9/4/834/s1. Figure S1: HBV infection of human liver chimeric mice. Figure S2: HBx recovered in CRL4^HBV^. Figure S3: Epitope-tagged DCAFs and HBx co-IP with myc-CUL4A. Figure S4: HBx does not compete with DCAF CRBN for binding to DDB1 in the CRL4. Table S1: Antibodies used in present study. Table S2: Restriction factor shRNAs used in current study. Table S3: Total proteins in CRL4^UN^ and CRL4^HBV^. Table S4: Proteins unique to CRL4^UN^ and CRL4^HBV^. Table S5: Proteins enriched in CRL4^UN^ and CRL4^HBV^. Table S6: Validation of DCAF-DDB1 interaction.

## References

[1] World Health Organization Hepatitis B Fact Sheet. https://www.who.int/news-room/fact-sheets/detail/hepatitis-b.

[2] Terrault N.A., Lok A.S.F., McMahon B.J., Chang K.M., Hwang J.P., Jonas M.M., Brown R.S., Bzowej N.H., Wong J.B. (2018). Update on prevention, diagnosis, and treatment of chronic hepatitis B: AASLD 2018 hepatitis B guidance. Hepatology.

[3] Tsuge M., Hiraga N., Akiyama R., Tanaka S., Matsushita M., Mitsui F., Abe H., Kitamura S., Hatakeyama T., Kimura T. (2010). HBx protein is indispensable for development of viraemia in human hepatocyte chimeric mice. J. Gen. Virol..

[4] Lucifora J., Arzberger S., Durantel D., Belloni L., Strubin M., Levrero M., Zoulim F., Hantz O., Protzer U. (2011). Hepatitis B Virus X protein is essential to initiate and maintain virus replication after infection. J. Hepatol..

[5] Scaglioni P.P., Melegari M., Wands J.R. (1997). Posttranscriptional regulation of hepatitis B virus replication by the precore protein. J. Virol..

[6] Melegari M., Wolf S.K., Schneider R.J. (2005). Hepatitis B virus DNA replication is coordinated by core protein serine phosphorylation and HBx expression. J. Virol..

[7] Bouchard M.J., Schneider R.J. (2004). The enigmatic X gene of hepatitis B virus. J. Virol..

[8] Twu J.S., Schloemer R.H. (1987). Transcriptional trans-activating function of hepatitis B virus. J. Virol..

[9] Spandau D.F., Lee C.H. (1988). Trans-activation of viral enhancers by the hepatitis B virus X protein. J. Virol..

[10] Belloni L., Pollicino T., De Nicola F., Guerrieri F., Raffa G., Fanciulli M., Raimondo G., Levrero M. (2009). Nuclear HBx binds the HBV minichromosome and modifies the epigenetic regulation of cccDNA function. Proc. Natl. Acad. Sci. USA.

[11] Hensel K.O., Cantner F., Bangert F., Wirth S., Postberg J. (2018). Episomal HBV persistence within transcribed host nuclear chromatin compartments involves HBx. Epigenet. Chromatin.

[12] Van de Klundert M.A., van den Biggelaar M., Kootstra N.A., Zaaijer H.L. (2016). Hepatitis B Virus Protein X Induces Degradation of Talin-1. Viruses.

[13] Decorsiere A., Mueller H., van Breugel P.C., Abdul F., Gerossier L., Beran R.K., Livingston C.M., Niu C., Fletcher S.P., Hantz O. (2016). Hepatitis B virus X protein identifies the Smc5/6 complex as a host restriction factor. Nature.

[14] Murphy C.M., Xu Y., Li F., Nio K., Reszka-Blanco N., Li X., Wu Y., Yu Y., Xiong Y., Su L. (2016). Hepatitis B Virus X Protein Promotes Degradation of SMC5/6 to Enhance HBV Replication. Cell Rep..

[15] Wu Z.J., Zhu Y., Huang D.R., Wang Z.Q. (2010). Constructing the HBV-human protein interaction network to understand the relationship between HBV and hepatocellular carcinoma. J. Exp. Clin. Cancer Res..

[16] Slagle B.L., Bouchard M.J. (2016). Hepatitis B Virus X and Regulation of Viral Gene Expression. Cold Spring Harb. Perspect. Med..

[17] Neuveut C., Wei Y., Buendia M.A. (2010). Mechanisms of HBV-related hepatocarcinogenesis. J. Hepatol..

[18] Benhenda S., Cougot D., Buendia M.A., Neuveut C. (2009). Hepatitis B virus X protein molecular functions and its role in virus life cycle and pathogenesis. Adv. Cancer Res..

[19] Minor M.M., Slagle B.L. (2014). Hepatitis B virus HBx protein interactions with the ubiquitin proteasome system. Viruses.

[20] Barry M., Fruh K. (2006). Viral modulators of Cullin RING Ubiquitin Ligases: Culling the host defense. Sci. STKE.

[21] Randow F., Lehner P.J. (2009). Viral avoidance and exploitation of the ubiquitin system. Nat. Cell Biol..

[22] Gustin J.K., Moses A.V., Fruh K., Douglas J.L. (2011). Viral takeover of the host ubiquitin system. Front. Microbiol..

[23] Gao G., Luo H. (2006). The ubiquitin-proteasome pathway in viral infections. Can. J. Physiol. Pharmacol..

[24] Lee T.-H., Elledge S.J., Butel J.S. (1995). Hepatitis B virus X protein interacts with a probable cellular DNA repair protein. J. Virol..

[25] Sitterlin D., Lee T.H., Prigent S., Tiollais P., Butel J.S., Transy C. (1997). Interaction of the UV-damaged DNA-binding protein with hepatitis B virus X protein is conserved among mammalian hepadnaviruses and restricted to transactivation-proficient X-insertion mutants. J. Virol..

[26] Li T., Robert E.I., van Breugel P.C., Strubin M., Zheng N. (2010). A promiscuous alpha-helical motif anchors viral hijackers and substrate receptors to the CUL4-DDB1 ubiquitin ligase machinery. Nat. Struct. Mol. Biol..

[27] Sitterlin D., Bergametti F., Tiollais P., Tennant B.C., Transy C. (2000). Correct binding of viral X protein to UVDDB-p127 cellular protein is critical for efficient infection by hepatitis B viruses. Oncogene.

[28] Leupin O., Bontron S., Schaeffer C., Strubin M. (2005). Hepatitis B virus X protein stimulates viral genome replication via a DDB1-dependent pathway distinct from that leading to cell death. J. Virol..

[29] Hodgson A.J., Hyser J.M., Keasler V.V., Cang Y., Slagle B.L. (2012). Hepatitis B virus regulatory HBx protein binding to DDB1 is required but is not sufficient for maximal HBV replication. Virology.

[30] He Y.J., McCall C.M., Hu J., Zeng Y., Xiong Y. (2006). DDB1 functions as a linker to recruit receptor WD40 proteins to CUL4-ROC1 ubiquitin ligases. Genes Dev..

[31] Higa L.A., Wu M., Ye T., Kobayashi R., Sun H., Zhang H. (2006). CUL4-DDB1 ubiquitin ligase interacts with multiple WD40-repeat proteins and regulates histone methylation. Nat. Cell Biol..

[32] Angers S., Li T., Yi X., MacCoss M.J., Moon R.T., Zheng N. (2006). Molecular architecture and assembly of the DDB1-CUL4A ubiquitin ligase machinery. Nature.

[33] Lee J., Zhou P. (2007). DCAFs, the missing link of the Cul4-DDB1 Ubiquitin Ligase. Mol. Cell.

[34] Keasler V.V., Slagle B.L., Kobarg J. (2008). The interaction of HBx with cellular DDB1. The Pleiotropic Functions of the Viral Protein HBx in Hepatitis B Virus Infection and the Development of Liver Cancer.

[35] Bontron S., Lin-Marq N., Strubin M. (2002). Hepatitis B virus X protein associated with UV-DDB1 induces cell death in the nucleus and is functionally antagonized by UV-DDB2. J. Biol. Chem..

[36] Ohta T., Michel J.J., Schottelius A.J., Xiong Y. (1999). ROC1, a homolog of APC11, represents a family of cullin partners with an associated ubiquitin ligase activity. Mol. Cell.

[37] Slagle B.L., Bouchard M.J. (2018). Role of HBx in hepatitis B virus persistence and its therapeutic implications. Curr. Opin. Virol..

[38] Mahon C., Krogan N.J., Craik C.S., Pick E. (2014). Cullin E3 ligases and their rewiring by viral factors. Biomolecules.

[39] Sarikas A., Hartmann T., Pan Z.Q. (2011). The cullin protein family. Genome Biol..

[40] Ulane C.M., Rodriguez J.J., Parisien J.P., Horvath C.M. (2003). STAT3 ubiquitylation and degradation by mumps virus suppress cytokine and oncogene signaling. J. Virol..

[41] Precious B., Childs K., Fitzpatrick-Swallow V., Goodbourn S., Randall R.E. (2005). Simian virus 5 V protein acts as an adaptor, linking DDB1 to STAT2, to facilitate the ubiquitination of STAT1. J. Virol..

[42] Le Rouzic E., Belaidouni N., Estrabaud E., Morel M., Rain J.C., Transy C., Margottin-Goguet F. (2007). HIV1 Vpr arrests the cell cycle by recruiting DCAF1/VprBP, a receptor of the Cul4-DDB1 ubiquitin ligase. Cell Cycle.

[43] Schrofelbauer B., Yu Q., Zeitlin S.G., Landau N.R. (2005). Human immunodeficiency virus type 1 Vpr induces the degradation of the UNG and SMUG uracil-DNA glycosylases. J. Virol..

[44] Hart S.N., Li Y., Nakamoto K., Subileau E.A., Steen D., Zhong X.B. (2010). A comparison of whole genome gene expression profiles of HepaRG cells and HepG2 cells to primary human hepatocytes and human liver tissues. Drug Metab. Dispos..

[45] Dandri M., Burda M.R., Torok E., Pollok J.M., Iwanska A., Sommer G., Rogiers X., Rogler C.E., Gupta S., Will H. (2001). Repopulation of mouse liver with human hepatocytes and in vivo infection with hepatitis B virus. Hepatology.

[46] Mercer D.F., Schiller D.E., Elliott J.F., Douglas D.N., Hao C., Rinfret A., Addison W.R., Fischer K.P., Churchill T.A., Lakey J.R. (2001). Hepatitis C virus replication in mice with chimeric human livers. Nat. Med..

[47] Bissig K.D., Le T.T., Woods N.B., Verma I.M. (2007). Repopulation of adult and neonatal mice with human hepatocytes: A chimeric animal model. Proc. Natl. Acad. Sci. USA.

[48] Bissig K.D., Wieland S.F., Tran P., Isogawa M., Le T.T., Chisari F.V., Verma I.M. (2010). Human liver chimeric mice provide a model for hepatitis B and C virus infection and treatment. J. Clin. Investig..

[49] Ulane C.M., Horvath C.M. (2002). Paramyxoviruses SV5 and HPIV2 assemble STAT protein ubiquitin ligase complexes from cellular components. Virology.

[50] Hrecka K., Gierszewska M., Srivastava S., Kozaczkiewicz L., Swanson S.K., Florens L., Washburn M.P., Skowronski J. (2007). Lentiviral Vpr usurps Cul4-DDB1[VprBP] E3 ubiquitin ligase to modulate cell cycle. Proc. Natl. Acad. Sci. USA.

[51] Schrofelbauer B., Hakata Y., Landau N.R. (2007). HIV-1 Vpr function is mediated by interaction with the damage-specific DNA-binding protein DDB1. Proc. Natl. Acad. Sci. USA.

[52] Srivastava S., Swanson S.K., Manel N., Florens L., Washburn M.P., Skowronski J. (2008). Lentiviral Vpx accessory factor targets VprBP/DCAF1 substrate adaptor for cullin 4 E3 ubiquitin ligase to enable macrophage infection. PLoS Pathog..

[53] Kang X., Chen X., He Y., Guo D., Guo L., Zhong J., Shu H.B. (2013). DDB1 is a cellular substrate of NS3/4A protease and required for hepatitis C virus replication. Virology.

[54] Liang X.Z., Pickering M.T., Cho N.H., Chang H., Volkert M.R., Kowalik T.F., Jung J.U. (2006). Deregulation of DNA damage signal transduction by herpesvirus latency-associated M2. J. Virol..

[55] Gastaldello S., Hildebrand S., Faridani O., Callegari S., Palmkvist M., Di G.C., Masucci M.G. (2010). A deneddylase encoded by Epstein-Barr virus promotes viral DNA replication by regulating the activity of cullin-RING ligases. Nat. Cell Biol..

[56] Vasilenko N.L., Snider M., Labiuk S.L., Lobanov V.A., Babiuk L.A., van Drunen Littel-van den Hurk S. (2012). Bovine herpesvirus-1 VP8 interacts with DNA damage binding protein-1 (DDB1) and is monoubiquitinated during infection. Virus Res..

[57] Trilling M., Le V.T., Fiedler M., Zimmermann A., Bleifuss E., Hengel H. (2011). Identification of DNA-damage DNA-binding protein 1 as a conditional essential factor for cytomegalovirus replication in interferon-gamma-stimulated cells. PLoS Pathog..

[58] Azuma H., Paulk N., Ranade A., Dorrell C., Al Dhalimy M., Ellis E., Strom S., Kay M.A., Finegold M., Grompe M. (2007). Robust expansion of human hepatocytes in Fah-/-/Rag2-/-/Il2rg-/- mice. Nat. Biotechnol..

[59] Keasler V.V., Hodgson A.J., Madden C.R., Slagle B.L. (2007). Enhancement of hepatitis B virus replication by the regulatory X protein in vitro and in vivo. J. Virol..

[60] Sechi S., Chait B.T. (1998). Modification of cysteine residues by alkylation. A tool in peptide mapping and protein identification. Anal. Chem..

[61] O’Leary N.A., Wright M.W., Brister J.R., Ciufo S., Haddad D., McVeigh R., Rajput B., Robbertse B., Smith-White B., Ako-Adjei D. (2016). Reference sequence (RefSeq) database at NCBI: Current status, taxonomic expansion, and functional annotation. Nucleic Acids Res..

[62] Schwanhausser B., Busse D., Li N., Dittmar G., Schuchhardt J., Wolf J., Chen W., Selbach M. (2011). Global quantification of mammalian gene expression control. Nature.

[63] Becker S.A., Lee T.H., Butel J.S., Slagle B.L. (1998). Hepatitis B virus X protein interferes with cellular DNA repair. J. Virol..

[64] Lin-Marq N., Bontron S., Leupin O., Strubin M. (2001). Hepatitis B virus X protein interferes with cell viability through interaction with the p127-kDa UV-damaged DNA-binding protein. Virology.

[65] Yan R., Zhang Y., Cai D., Liu Y., Cuconati A., Guo H. (2015). Spinoculation Enhances HBV Infection in NTCP-Reconstituted Hepatocytes. PLoS ONE.

[66] Schulze A., Schieck A., Ni Y., Mier W., Urban S. (2010). Fine mapping of pre-S sequence requirements for hepatitis B virus large envelope protein-mediated receptor interaction. J. Virol..

[67] Ni Y., Lempp F.A., Mehrle S., Nkongolo S., Kaufman C., Falth M., Stindt J., Koniger C., Nassal M., Kubitz R. (2014). Hepatitis B and D viruses exploit sodium taurocholate co-transporting polypeptide for species-specific entry into hepatocytes. Gastroenterology.

[68] Soucy T.A., Smith P.G., Milhollen M.A., Berger A.J., Gavin J.M., Adhikari S., Brownell J.E., Burke K.E., Cardin D.P., Critchley S. (2009). An inhibitor of NEDD8-activating enzyme as a new approach to treat cancer. Nature.

[69] Sharma P., Nag A. (2014). CUL4A ubiquitin ligase: A promising drug target for cancer and other human diseases. Open Biol..

[70] Qian M.X., Pang Y., Liu C.H., Haratake K., Du B.Y., Ji D.Y., Wang G.F., Zhu Q.Q., Song W., Yu Y. (2013). Acetylation-mediated proteasomal degradation of core histones during DNA repair and spermatogenesis. Cell.

[71] Mandemaker I.K., Geijer M.E., Kik I., Bezstarosti K., Rijkers E., Raams A., Janssens R.C., Lans H., Hoeijmakers J.H., Demmers J.A. (2018). DNA damage-induced replication stress results in PA200-proteasome-mediated degradation of acetylated histones. EMBO Rep..

[72] Pollicino T., Belloni L., Raffa G., Pediconi N., Squadrito G., Raimondo G., Levrero M. (2006). Hepatitis B virus replication is regulated by the acetylation status of hepatitis B virus cccDNA-bound H3 and H4 histones. Gastroenterology.

[73] He Q., Li W., Ren J., Huang Y., Huang Y., Hu Q., Chen J., Chen W. (2016). ZEB2 inhibits HBV transcription and replication by targeting its core promoter. Oncotarget.

[74] Liou J., Kim M.L., Heo W.D., Jones J.T., Myers J.W., Ferrell J.E., Meyer T. (2005). STIM is a Ca2+ sensor essential for Ca2+-store-depletion-triggered Ca2+ influx. Curr. Biol..

[75] Bouchard M.J., Wang L.-H., Schneider R.J. (2002). Calcium signaling by HBx protein in hepatitis B virus DNA replication. Science.

[76] Bouchard M.J., Puro R.J., Wang L., Schneider R.J. (2003). Activation and inhibition of cellular calcium and tyrosine kinase signaling pathways identify targets of the HBx protein involved in hepatitis B virus replication. J. Virol..

[77] McCarthy M.K., Weinberg J.B. (2015). The immunoproteasome and viral infection: A complex regulator of inflammation. Front. Microbiol..

[78] Bergametti F., Sitterlin D., Transy C. (2002). Turnover of hepatitis B virus X protein is regulated by damaged DNA-binding complex. J. Virol..

[79] Yan H., Zhong G., Xu G., He W., Jing Z., Gao Z., Huang Y., Qi Y., Peng B., Wang H. (2012). Sodium taurocholate cotransporting polypeptide is a functional receptor for human hepatitis B and D virus. Elife.

[80] Ju L., Wing J., Taylor E., Brandt R., Slijepcevic P., Horsch M., Rathkolb B., Racz I., Becker L., Hans W. (2013). SMC6 is an essential gene in mice, but a hypomorphic mutant in the ATPase domain has a mild phenotype with a range of subtle abnormalities. DNA Repair (Amst).

[81] Uhlen M., Oksvold P., Fagerberg L., Lundberg E., Jonasson K., Forsberg M., Zwahlen M., Kampf C., Wester K., Hober S. (2010). Towards a knowledge-based Human Protein Atlas. Nat. Biotechnol..

[82] Uhlen M., Fagerberg L., Hallstrom B.M., Lindskog C., Oksvold P., Mardinoglu A., Sivertsson A., Kampf C., Sjostedt E., Asplund A. (2015). Proteomics. Tissue-based map of the human proteome. Science.

[83] Leupin O., Bontron S., Strubin M. (2003). Hepatitis B virus X protein and simian virus 5 V protein exhibit, similar UV-DDB1 binding properties to mediate distinct activities. J. Virol..

[84] Djakbarova U., Marzluff W.F., Koseoglu M.M. (2016). DDB1 and CUL4 associated factor 11 (DCAF11) mediates degradation of Stem-loop binding protein at the end of S phase. Cell Cycle.

[85] Brodersen M.M., Lampert F., Barnes C.A., Soste M., Piwko W., Peter M. (2016). CRL4(WDR23)-Mediated SLBP Ubiquitylation Ensures Histone Supply during DNA Replication. Mol. Cell.

[86] Li G., Ji T., Chen J., Fu Y., Hou L., Feng Y., Zhang T., Song T., Zhao J., Endo Y. (2017). CRL4(DCAF8) Ubiquitin Ligase Targets Histone H3K79 and Promotes H3K9 Methylation in the Liver. Cell Rep..

[87] Klein C.J., Wu Y., Vogel P., Goebel H.H., Bonnemann C., Zukosky K., Botuyan M.V., Duan X., Middha S., Atkinson E.J. (2014). Ubiquitin ligase defect by DCAF8 mutation causes HMSN2 with giant axons. Neurology.

[88] Fischer E.S., Bohm K., Lydeard J.R., Yang H., Stadler M.B., Cavadini S., Nagel J., Serluca F., Acker V., Lingaraju G.M. (2014). Structure of the DDB1-CRBN E3 ubiquitin ligase in complex with thalidomide. Nature.

[89] Kataoka K., Nakamura C., Asahi T., Sawamura N. (2016). Mitochondrial cereblon functions as a Lon-type protease. Sci. Rep..

[90] Xing Z., Tang X., Gao Y., Da L., Song H., Wang S., Tiollais P., Li T., Zhao M. (2011). The human LIS1 is downregulated in hepatocellular carcinoma and plays a tumor suppressor function. Biochem. Biophys. Res. Commun..

[91] Li X., Liu L., Li R., Wu A., Lu J., Wu Q., Jia J., Zhao M., Song H. (2018). Hepatic loss of Lissencephaly 1 (Lis1) induces fatty liver and accelerates liver tumorigenesis in mice. J. Biol. Chem..

[92] McKenney R.J., Vershinin M., Kunwar A., Vallee R.B., Gross S.P. (2010). LIS1 and NudE induce a persistent dynein force-producing state. Cell.

[93] Huang J., Roberts A.J., Leschziner A.E., Reck-Peterson S.L. (2012). Lis1 acts as a “clutch” between the ATPase and microtubule-binding domains of the dynein motor. Cell.

[94] Lam C., Vergnolle M.A., Thorpe L., Woodman P.G., Allan V.J. (2010). Functional interplay between LIS1, NDE1 and NDEL1 in dynein-dependent organelle positioning. J. Cell Sci..

[95] Egan M.J., Tan K., Reck-Peterson S.L. (2012). Lis1 is an initiation factor for dynein-driven organelle transport. J. Cell Biol..

[96] Alqarni S.S., Murthy A., Zhang W., Przewloka M.R., Silva A.P., Watson A.A., Lejon S., Pei X.Y., Smits A.H., Kloet S.L. (2014). Insight into the architecture of the NuRD complex: Structure of the RbAp48-MTA1 subcomplex. J. Biol. Chem..

[97] Goto T., Matsuzawa J., Iemura S., Natsume T., Shibuya H. (2016). WDR26 is a new partner of Axin1 in the canonical Wnt signaling pathway. FEBS Lett..

[98] Slagle B.L., Lee T.-H., Medina D., Finegold M.J., Butel J.S. (1996). Increased sensitivity to the hepatocarcinogen diethylnitrosamine in transgenic mice carrying the hepatitis B virus X gene. Mol. Carcinog..

[99] Dandri M., Schirmacher P., Rogler C.E. (1996). Woodchuck hepatitis virus X protein is present in chronically infected woodchuck liver and woodchuck hepatocellular carcinomas which are permissive for viral replication. J. Virol..

[100] Livingston C.M., Ramakrishnan D., Strubin M., Fletcher S.P., Beran R.K. (2017). Identifying and Characterizing Interplay between Hepatitis B Virus X Protein and Smc5/6. Viruses.

[101] Petroski M.D., Deshaies R.J. (2005). Function and regulation of Cullin-RING ubiquitin ligases. Nat. Rev. Mol. Cell Biol..

[102] Sekiba K., Otsuka M., Ohno M., Yamagami M., Kishikawa T., Seimiya T., Suzuki T., Tanaka E., Ishibashi R., Funato K. (2019). Pevonedistat, a Neuronal Precursor Cell-Expressed Developmentally Down-Regulated Protein 8-Activating Enzyme Inhibitor, Is a Potent Inhibitor of Hepatitis B Virus. Hepatology.

[103] Aragon L. (2018). The Smc5/6 Complex: New and Old Functions of the Enigmatic Long-Distance Relative. Annu. Rev. Genet..

[104] Niu C., Livingston C.M., Li L., Beran R.K., Daffis S., Ramakrishnan D., Burdette D., Peiser L., Salas E., Ramos H. (2017). The Smc5/6 Complex Restricts HBV when Localized to ND10 without Inducing an Innate Immune Response and Is Counteracted by the HBV X Protein Shortly after Infection. PLoS ONE.

[105] Abdul F., Filleton F., Gerossier L., Paturel A., Hall J., Strubin M., Etienne L. (2018). Smc5/6 Antagonism by HBx Is an Evolutionarily Conserved Function of Hepatitis B Virus Infection in Mammals. J. Virol..

[106] Wang Y., Li Y. (2018). miR-146 promotes HBV replication and expression by targeting ZEB2. Biomed. Pharmacother..

[107] Li J.F., Dai X.P., Zhang W., Sun S.H., Zeng Y., Zhao G.Y., Kou Z.H., Guo Y., Yu H., Du L.Y. (2015). Upregulation of microRNA-146a by hepatitis B virus X protein contributes to hepatitis development by downregulating complement factor H. MBio.

[108] Cougot D., Allemand E., Riviere L., Benhenda S., Duroure K., Levillayer F., Muchardt C., Buendia M.A., Neuveut C. (2012). Inhibition of PP1 phosphatase activity by HBx: A mechanism for the activation of hepatitis B virus transcription. Sci. Signal..

[109] Benhenda S., Ducroux A., Riviere L., Sobhian B., Ward M.D., Dion S., Hantz O., Protzer U., Michel M.L., Benkirane M. (2013). Methyltransferase PRMT1 is a binding partner of HBx and a negative regulator of hepatitis B virus transcription. J. Virol..

[110] Zhang W., Chen J., Wu M., Zhang X., Zhang M., Yue L., Li Y., Liu J., Li B., Shen F. (2017). PRMT5 restricts hepatitis B virus replication through epigenetic repression of covalently closed circular DNA transcription and interference with pregenomic RNA encapsidation. Hepatology.

[111] Belloni L., Allweiss L., Guerrieri F., Pediconi N., Volz T., Pollicino T., Petersen J., Raimondo G., Dandri M., Levrero M. (2012). IFN-alpha inhibits HBV transcription and replication in cell culture and in humanized mice by targeting the epigenetic regulation of the nuclear cccDNA minichromosome. J. Clin. Investig..

[112] Cheng W., Chen G., Jia H., He X., Jing Z. (2018). DDX5 RNA Helicases: Emerging Roles in Viral Infection. Int. J. Mol. Sci..

[113] Zhang H., Xing Z., Mani S.K., Bancel B., Durantel D., Zoulim F., Tran E.J., Merle P., Andrisani O. (2016). RNA helicase DEAD box protein 5 regulates Polycomb repressive complex 2/Hox transcript antisense intergenic RNA function in hepatitis B virus infection and hepatocarcinogenesis. Hepatology.

[114] Kim J.S., Rho B., Lee T.H., Lee J.M., Kim S.J., Park J.H. (2006). The interaction of hepatitis B virus X protein and protein phosphatase type 2 Calpha and its effect on IL-6. Biochem. Biophys. Res. Commun..

[115] Nishitsuji H., Ujino S., Harada K., Shimotohno K. (2018). TIP60 Complex Inhibits Hepatitis B Virus Transcription. J. Virol..

